# Evolutionary history of the extant amphioxus lineage with shallow-branching diversification

**DOI:** 10.1038/s41598-017-00786-5

**Published:** 2017-04-25

**Authors:** Takeshi Igawa, Masafumi Nozawa, Daichi G. Suzuki, James D. Reimer, Arseniy R. Morov, Yiquan Wang, Yasuhisa Henmi, Kinya Yasui

**Affiliations:** 1 Amphibian Research Center, Hiroshima University, 1-3-1 Kagamiyama, Higashi-hiroshima, Hiroshima 739-8529 Japan; 2 Department of Biological Sciences, School of Science and Engineering, Tokyo Metropolitan University, 1-1 Minamiosawa, Hachioji, Tokyo 192-0397 Japan; 3 Department of Biological Sciences, Graduate School of Life and Environmental Sciences, Tsukuba University, 1-1-1 Tennodai, Tsukuba, Ibaraki 305-8572 Japan; 4 Department of Biology, Chemistry & Marine Sciences, Faculty of Science, University of the Ryukyus, 1 Senbaru, Nishihara, Okinawa 903-0213 Japan; 5 Department of Biological Science, Graduate School of Science, Hiroshima University, 1-3-1 Kagamiyama, Higashi-hiroshima, Hiroshima 739-8526 Japan; 6 Depertment of Zoology and General Biology, Institute of Fundamental Medicine and Biology, Kazan (Volga Region) Federal University, 18 Kremlyovskaya St., Kazan, 420008 Republic of Tatarstan Russian Federation; 7 State Key Laboratory of Cellular Stress Biology, School of Life Sciences, Xiamen University, Xiamen, 361102 China; 8 Aitsu Marine Station, Center for Marine Environmental Studies, Kumamoto University, 6061 Aitsu, Kami-Amakusa, Kumamoto 861-6102 Japan; 9 Nobel Institute for Neurophysiology, Department of Neuroscience, Karolinska Institutet, SE-171 77 Stockholm, Sweden

## Abstract

Amphioxus or lancelets have been regarded as a key animal in understanding the origin of vertebrates. However, the evolutionary history within this lineage remains unexplored. As the amphioxus lineage has likely been separated from other chordates for a very long time and displays a marked left-right asymmetry, its evolutionary history is potentially helpful in better understanding chordate and vertebrate origins. We studied the phylogenetic relationships within the extant amphioxus lineage based on mitochondrial genomes incorporating new *Asymmetron* and *Epigonichthys* populations, and based on previously reported nuclear transcriptomes. The resulting tree patterns are consistent, showing the *Asymmetron* clade diverging first, followed by the *Epigonichthys* and *Branchiostoma* clades splitting. Divergence time estimates based on nuclear transcriptomes with vertebrate calibrations support a shallow diversification of the extant amphioxus lineage in the Tertiary. These estimates fit well with the closure of seaways between oceans by continental drift, ocean currents, and present geographical distributions, and suggest a long cryptic history from the origin of amphioxus to its most recent diversification. Deduced character polarities based on phylogenetic analyses suggest that the common ancestor of the extant amphioxus existed in a tiny epibenthic state with larva-like appearance of extant amphioxus, likely with ciliate epidermis.

## Introduction

Among chordates, the deepest branching chordate subphylum Cephalochordata displays a unique asymmetrical development^1^. Cephalochordata is a small group divided into three genera, *Asymmetron*, *Branchiostoma*, and *Epigonichthys* ^2–6^, and comprises about 30 species at present^7^. All extant species are generally inactive suspension feeders living in sand substrata (e.g. *Branchiostoma* ^8^). Curiously, however, all species display streamlined appearances similar to actively swimming fish, and do not develop a mucus layer or cuticles on the skin unlike in many other sand dwellers. These incongruous features of amphioxus provide clues for understanding the origin of the chordate lineage. To reconstruct an accurate image of the last common ancestor (LCA) of the chordate lineage, it is essential to understand the LCA of the amphioxus lineage by elucidating character polarities from varying features found in living amphioxus species based on reliable molecular phylogenetic analyses.

A recent genome-based phylogenetic study suggests that the Cephalochordata appeared in the Precambrian, soon after the split of bilaterian animals into deuterostomes and protostomes^9^. The branching order of the three extant amphioxus genera was also suggested based on the mitochondrial genomic (mitogenomic) sequences of *Asymmetron* and *Epigonichthys* specimens from the Maldives, Bermuda, and western Japan^3, 5^; with the genus *Asymmetron* diverging first followed by *Epigonichthys* and *Branchiostoma*. This phylogenetic framework is important for understanding the origin of chordates as the amphioxus clades that develop gonads only on the right side (*Asymmetron*, *Epigonichthys*) are not monophyletic. However, considering the wide distribution of amphioxus genera, each possibly including cryptic species^4^, there is a need to expand the phylogenetic dataset utilised in reconstruction of the evolutionary history of the amphioxus lineage.

Divergence times for the amphioxus lineage have also been previously proposed^3–5, 10^. Some of these studies applied deep reference points such as 652 Ma and 891 Ma^5^, but these are inappropriate for mitogenomes due to substitution saturation^11, 12^, while other studies have depended exclusively on outgroup reference points and have estimated divergence of *Asymmetron* in the Mesozoic or Paleozoic (120–360 Ma) with a very wide upper confidence limit that reaches to ~25 Ma in the Cenozoic^5, 10^. These studies accordingly suggest very slow evolution within the amphioxus lineage. However, a whole genome study on *Branchiostoma belcheri* found that in this species gene turnover was active and also that the amino acid substitution rate was comparable to that of vertebrates with rapid substitutions^13^. Thus, although the evolutionary rate of the amphioxus lineage has been regarded as being slow, a re-evaluation of this theory is clearly needed due to uncertainty.

Northwest Pacific coastal waters surrounding China, Taiwan, and western Japan harbour six or more amphioxus species, including representatives of all three genera^2, 14–17^; *Asymmetron lucayanum* Andrews, 1893, *A. inferum* Nishikawa, 2004, *Epigonichthys maldivensis* (Forster Cooper, 1903), *E. cultellus* Peters, 1877, *Branchiostoma belcheri* (Gray, 1847), and *B. japonicum* (Willey, 1897). *Asymmetron* and *Epigonichthys* are characterised by gonads that develop only on the right side of the body. *Asymmetron lucayanum* is currently thought to be distributed circumtropically in the Indian, Pacific, and Atlantic Oceans^7^, but based on analyses of sequences of *cytochrome c oxidase subunit I* (*coxI*), it has been suggested that this taxon contains at least three cryptic species^4^. Around Kuroshima Island, Okinawa, Japan, *A. lucayanum* and *E. maldivensis* have been reported as occurring sympatrically, and the Kuroshima *A. lucayanum* population has been suggested to contain two cryptic species^3, 4^. In Taiwan, *A. lucayanum* and *E. maldivensis* are sympatric in most habitats as seen at Kuroshima Island^14, 16^. *Epigonichthys cultellus* has been reported from the northern South China Sea^16, 18–20^. Although all of these amphioxus species with dextral gonads are distributed in tropical or subtropical waters, undescribed *Epigonichthys*-like amphioxus specimens were collected in this study from temperate Kyushu Island, Japan.

Whole mitochondrial DNA sequences allow comparisons among a wide range of populations and can serve as a useful tool for understanding phylogenetic relationships within the amphioxus lineage^3, 5^. In this study we performed mitogenomic analyses by utilising new data acquired from *Epigonichthys* and *Asymmetron* specimens from Taiwan, from one of the *Epigonichthys*-like specimens from Kyushu, Japan, and from *Asymmetron* species from the Bahamas, West Atlantic (the type locality of *A. lucayanum* ^21^), and combined our acquired data with all publically available amphioxus mitogenomic sequences. We also analysed a nuclear transcriptome-based phylogeny complied from publically available data that included *Branchiostoma*, *Asymmetron*, and vertebrate species. Furthermore, we estimated divergence times with an expanded ingroup dataset of nuclear transcriptomes with carefully selected calibration points, and then verified if the resulting estimates corresponded to any reliably dated or well-known isolating events.

This study found that contrary to previous studies^3–5, 10^ the extant amphioxus lineage has experienced rather recent diversification events in the Tertiary. Our divergence estimates are consistent with the genetic proximity between species observed within the *Asymmetron* clade as we found that nucleotide substitution rates in amphioxus genomes were comparable to mammalian rates. In this phylogenetic framework, we suggest that *Asymmetron lucayanum* should be divided into two species, *Asymmetron pelagicum* (Günter, 1889) (Pacific and West Atlantic clades in ref. 4) and *Asymmetron orientale* Parker, 1904 (Indian and West Pacific clade in ref. 4). Character distributions in the three genera, when based on the present phylogenetic analyses, suggest that the amphioxus crown lineage survived in a tiny epibenthos form for a long period from its origin to the onset of the most recent diversification.

## Results

### Phylogeny within the amphioxus lineage

**Figure 1:**
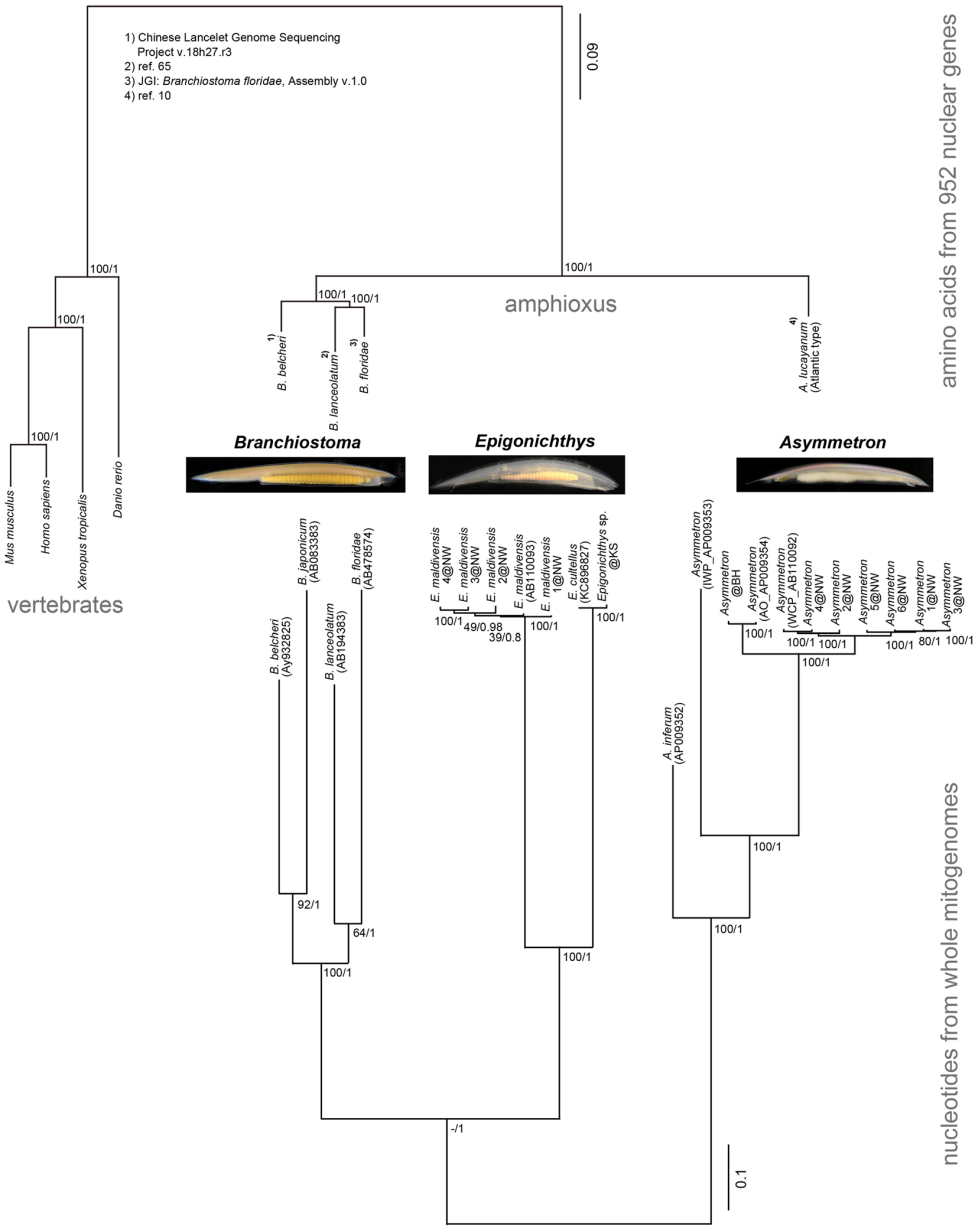
Phylogenetic trees of three extant amphioxus genera based on mitogenomes and nuclear transcriptomes. Bayesian and maximum likelihood methods gave the same topology for both trees based on amino acid sequences of nuclear transcriptomes (top) and on nucleotides of whole mitogenomes (bottom). Values at nodes are bootstrap values (left side) and posterior probabilities (right side). The top tree includes four vertebrate species and shows the monophyly of *B. lanceolatum* and *B. floridae* as shown in the bottom tree. The *Asymmetron* sequence in the top tree is derived from Atlantic type^4^. Numerals and initials in *Epigonichthys* and *Asymmetron* clades denote specimen numbers and types^4^/locations. IWP, Indo-West Pacific type; AO, Atlantic type; WCP, West-Central Pacific type; BH, Bahamas; NW, Nanwan Bay; KS, Kyushu.

Phylogenetic analyses based on the mitogenomes’ nucleotide (nt) sequences resulted in trees topologically identical between Bayesian inference (BI) and maximum likelihood (ML) methods (Fig. 1). The phylogeny confirmed that the *Asymmetron* clade first diverged from the ancestral *Epigonichthys* + *Branchiostoma* clade (Fig. 1), as proposed in previous studies^5, 17, 22^. Within the *Asymmetron* clade, *Asymmetron inferum*, which is found near whale falls^2^, was sister to the other groups. The Indian Ocean *Asymmetron* clade (specimen from the Maldives)^4^ then separated from a clade comprising specimens from Taiwan, Bermuda^4^, the Bahamas, and Okinawa^3^ (Fig. 1).

When we compared amino acid (aa) sequences, the branching pattern of the *Branchiostoma* clade was inconsistent between BI and ML analyses. The most probable tree resulting from ML was almost identical to that of nt, whereas the BI tree showed that *B. floridae* was sister to all other *Branchiostoma* species with low posterior probability (also see ref. 17).

Phylogenetic analyses based on aa sequences of 952 protein-coding genes deduced from nuclear transcriptomes of amphioxus and vertebrate species generally supported the mitogenomic analyses, although these analyses lacked *Epigonichthys* data, and the branching order within *Branchiostoma* showed a monophyletic relationship between *B. lanceolatum* and *B. floridae* in both BI and ML methods (Fig. 1).

### Inference of divergence times

**Figure 2:**
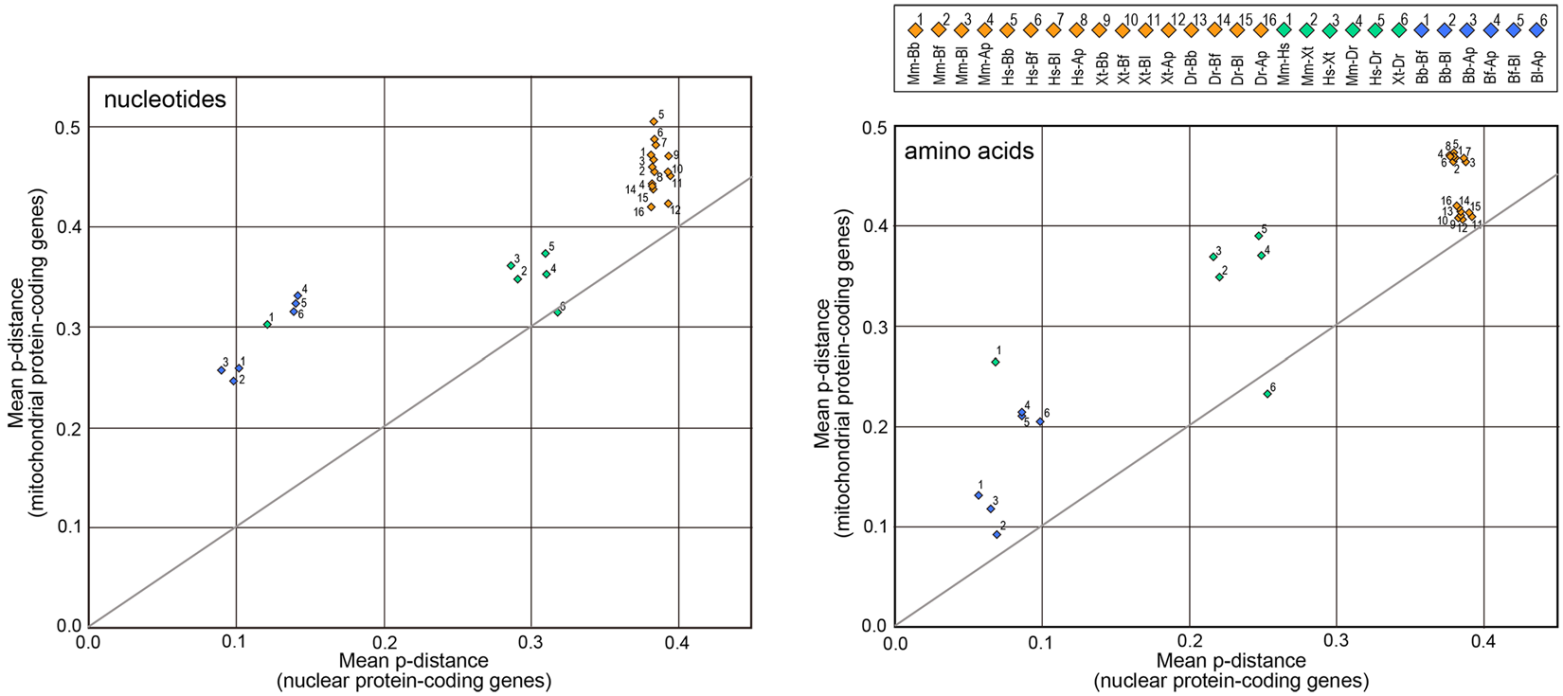
p-distances of mitochondrial and nuclear protein-coding genes between amphioxus and vertebrate taxa. Pairwise p-distances of nucleotide and amino acid sequences of 13 mitochondrial and 952 nuclear genes were calculated for all combinations of four amphioxus and four vertebrate species. The pairwise distances were calculated using MEGA-CC^71^ and plotted by mean values for all corresponding species pairs.

In estimates of divergence times within a single lineage, calibration time points should be ideally related to the ingroup history rather than inferred based on outgroup calibrations^23^. Unfortunately, however, there is no reliable calibration point within the amphioxus lineage, and we thus calculated pairwise nucleotide substitutions per unit nucleotide length (p-distance). Our results found that substitution rates in mitochondria and nuclear genes between amphioxus species are comparable to those between humans and mice, whose divergence is dated to 81 ± 10 Ma^24^ (Fig. 2). In the comparison between amphioxus species, the nucleotide substitution rate in mitochondrial genes was three times as high as that in nuclear genes, as seen in humans and mice. In higher vertebrate taxa and vertebrate-amphioxus comparisons, the substitution per unit length did not increase in mitochondrial genes unlike as seen in nuclear gene analyses, suggesting substitution saturation of mitochondrial genes (Fig. 2). In amino acid substitution analyses, saturation in mitochondrial genes between deep branching taxa was weaker than as seen in nucleotide substitution analyses. A comparison including mammalian taxa noticed faster substitutions in mitochondrial genes than in nuclear genes (Fig. 2). These substitution results have two possible meanings; either diversification of extant amphioxus started as far back in time as the human-mouse divergence (81 ± 10 Ma), or alternately that substitutions in the amphioxus lineage are slower than those in the vertebrate lineage, as has been previously suggested^10^.

**Figure 3:**
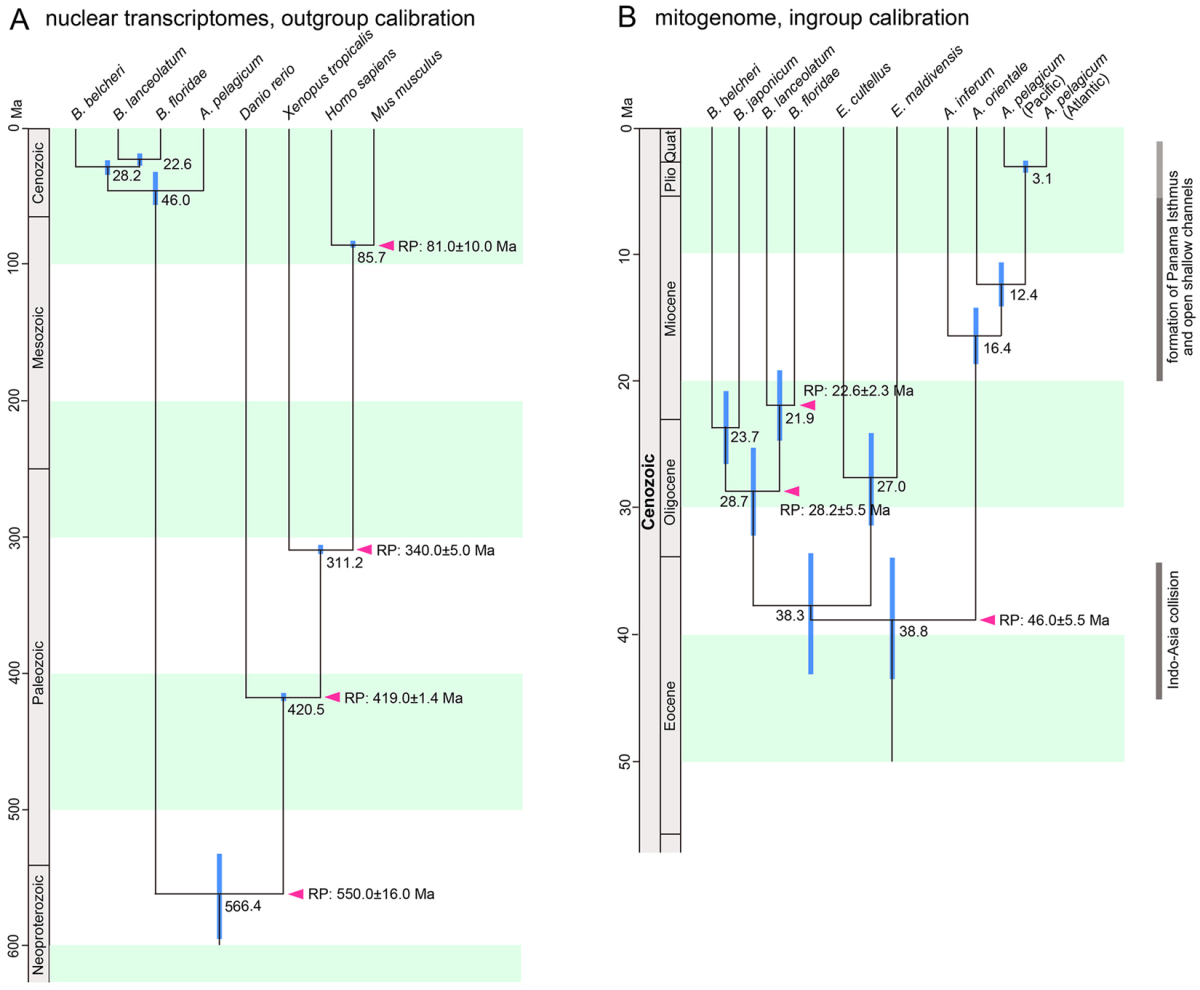
Divergence time estimates in amphioxus lineage. (**A**) Estimate based on nuclear transcriptome comparison calibrated with vertebrate fossil records^26^. Numerals at nodes denote estimated split time in Ma. (**B**) Estimate based on mitogenomic comparison with ingroup calibration points derived from (**A**). Timings of formation of the Isthmus of Panama^36^ and of Arabia-Eurasia^38^ and Indo-Asia collisions^72, 73^ are also shown. Blue bars denote 95% confidence interval.

We first estimated divergence times based on nuclear transcriptome data with four reference points; the amphioxus-vertebrates (550.0 ± 16.0 Ma^25^), ray-finned fish-tetrapods (419.0 ± 1.4 Ma^25^), amphibians-amniotes (340.0 ± 5.0 Ma^24^), and primates-rodents (81.0 ± 10.0 Ma^24^) splits. Fossil evidence for the earliest vertebrate, *Haikouichthys ercaicunensis* ^26^, is more reliable than disputable amphioxus-related fossils such as *Haikouella* spp.^27^ and *Pikaia gracilens* ^28^ as the reference point for the split of the amphioxus lineage from other chordates. In fact, as the latter fossils are the same age as or younger than the former, the choice of fossil actually does not affect the calibration of this reference point. Calculations showed two convergence regions of estimates depending on seed values; one converged at >100 Ma for the divergence of *Asymmetron*- (*Branchiostoma* + *Epigonichthys*) as in previous studies^3, 5, 10^, while the other converged at 46.0 (32.4–56.6) Ma for the same divergence. As the latter result gave a significantly higher mean Bayesian posterior probability summarizing three runs of each result [older divergence (lnL = −137011) vs. younger divergence (lnL = −137008) p < 3.3 × 10^−12^, Welch’s t-test after Bonferroni correction], we accepted the latter result. This estimate dated the divergence times between extant amphioxus clades as two- to four times younger than previous estimates (Fig. 3A)^5, 10^. When calibration points are set at deep nodes in mitogenome-based estimates, saturation of nucleotide substitution can lead to over-estimation of divergence timing^11^. We thus inferred divergence times in this study by using mitogenomes with reference points obtained from the nuclear transcriptome-based estimates of the *Asymmetron*-(*Branchiostoma* + *Epigonichthys*) (46.0 ± 5.5 Ma), *B. belcheri*-(*B. lanceolatum* + *B. floridae*) (28.2 ± 5.5 Ma), and *B. lanceolatum*-*B. floridae* (22.6 ± 2.3 Ma) splits. These analyses estimated the timing of the splits for *A. inferum* diverging from the remainder of *Asymmetron* at 16.4 Ma, for the divergence of the Indian *Asymmetron* population from the Pacific + Atlantic population at 12.4 Ma, and for divergence of the Atlantic and Pacific populations at 3.1 Ma (Fig. 3B). Our estimates based on the mitogenomes also showed splits into the three current extant genera during a short period, and more recent diversifications at the species-level within *Asymmetron* (Fig. 3B) than previously has been assumed.

The mitogenome-based phylogenetic analyses in this study showed that the Atlantic and Pacific *Asymmetron* populations were more closely related compared to the more distant Indian population (Fig. 1), as seen in a previous study^4^. We thus searched for isolation events that could explain this pattern, and based on our estimated divergence timings, we focused on the formation of the Isthmus of Panama and the closure of the Neo-Tethys Sea. We then again estimated divergence times within the amphioxus lineage based on the mitogenomic data utilizing these two geological reference points separately. We verified the congruence between estimated and given times, and then finally conducted an estimate with both reference points. With both reference points, the initial split of the amphioxus crown lineage into the *Asymmetron* and *Branchiostoma* + *Epigonichthys* clades was 42.2 (49.6–35.1) Ma, followed by the split of *Brachiostoma* and *Epigonichthys* at 35.6 (42.0–29.7) Ma (Supplementary Fig. S2). These estimates are consistent with our estimates obtained based on calibration points derived from nuclear transcriptome sequences (Fig. 3B).

### Mitogenomic properties and taxonomic identity of sympatric *Asymmetron* and *Epigonichthys* specimens from Taiwan

One species each of *Asymmetron* and *Epigonichthys* are sympatric in tropical Nanwan Bay at the southern tip of Taiwan, where they are found in fine sand substratum containing a considerable amount of mud at 10–15 m depths, as reported previously^14^. The annual seawater temperature (monthly means) ranges between 23–29 °C^29^, and there is an extensive coral reef ecosystem at this location^30^. We collected *Asymmetron* individuals measured approximately 10–15 mm in body length, and *Epigonichthys* individuals ranging from 10 to 25 mm from Nanwan Bay. Some individuals of both species were developing gonads in March and May 2014 and in May 2015.

**Figure 4:**
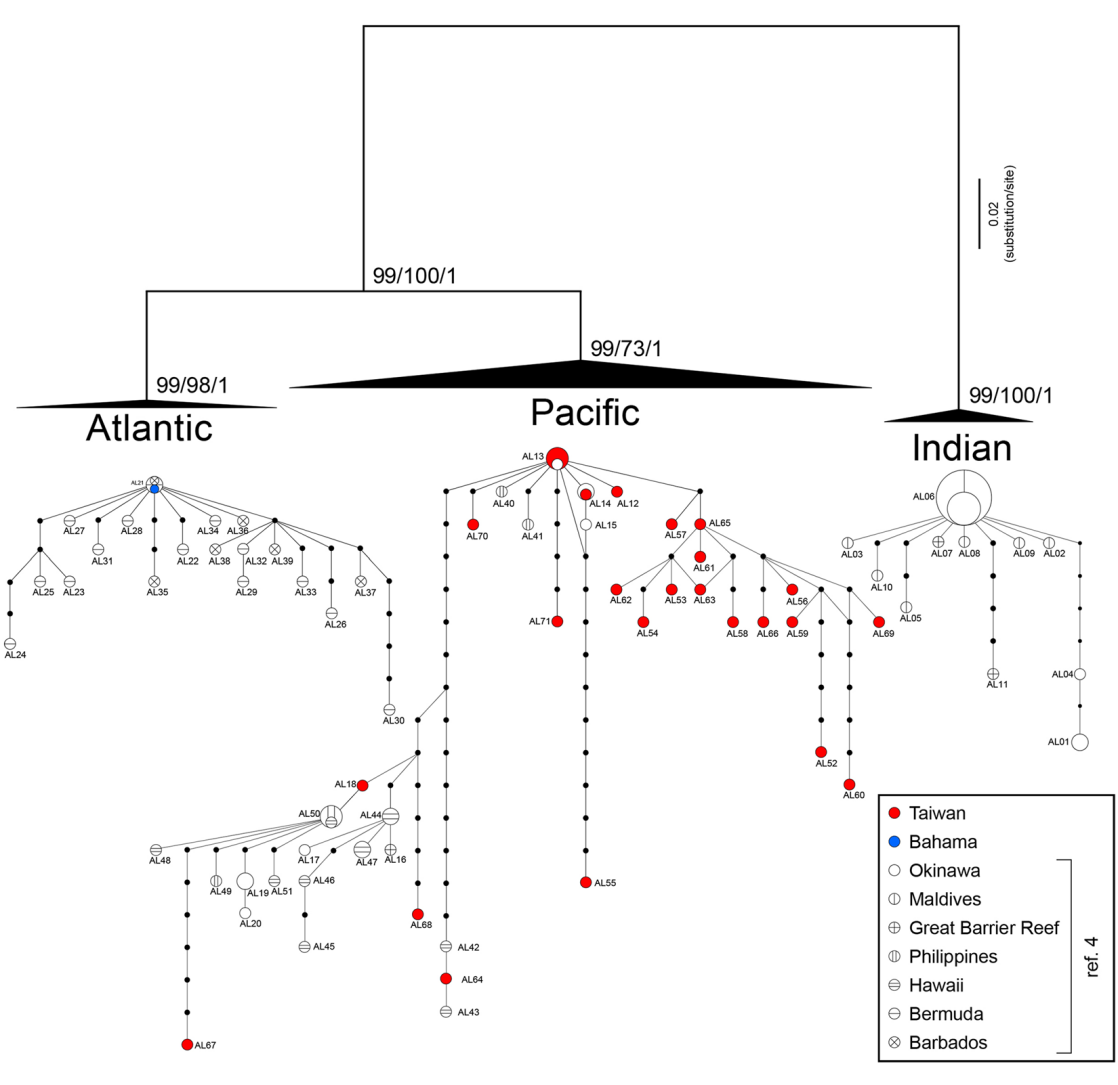
Genetic relationships of *Asymmetron* populations. Neighbour-joining, maximum likelihood, and Bayesian methods produced trees with the same topology for *cytochrome c oxidase subunit I* sequences (top) and show a close relationship between Pacific and Atlantic haplotype groups. Parsimony haplotype networks based on the same gene (bottom) show that haplotypes of all Taiwanese specimens are within the Pacific group. Sizes of circles indicate haplotype frequency and dots represent missing haplotypes.

Previously, based on *cox1* sequence analyses, two different clades of *A. lucayanum* were confirmed to exist sympatrically in Okinawa, Japan; these have been previously designated as ‘West-Central Pacific’ (herein Pacific type) and ‘Indo-West Pacific’ (herein Indian type) clades based on specimen origin^4^. We therefore compared sequences of *coxI* including all sequences in ref. 4 and found 21 unique haplotypes that include two previously reported^4^ within the Taiwanese population. All neighbour-joining, ML and BI trees based on *coxI* sequences showed the same branching pattern of Indian-(Pacific + Atlantic) (Fig. 4), which was also recovered in the whole mitogenomic sequence analyses (Fig. 1). In the network tree, all haplotypes from the Taiwanese population (n = 25) belonged to the Pacific type and was divided into two clusters; one contained haplotypes from the West Pacific while the other contained mainly haplotypes from Hawai’i (Fig. 4).

As all *Asymmetron* specimens from Nanwan Bay were included within the Pacific type, we sequenced the full-length of mitogenomes for six randomly selected *Asymmetron* specimens from Nanwan Bay: AL52 (1@NW), AL13 (2@NW), AL57 (3@NW), AL67 (4@NW), AL70 (5@NW), and AL71 (6@NW), as well as for one *Asymmetron* specimen from the Bahamas: AL21 (@BH). The gene orders were all identical to those previously reported for *A. lucayanum* ^3, 5^, but the lengths of nucleotide sequences slightly varied, from 15,095 to 15,097 bp (Accession Numbers AP015017-015023), due to indel sites in 12S rRNA, 16S rRNA, tRNA-Ile genes, and/or the control region.

**Figure 5:**
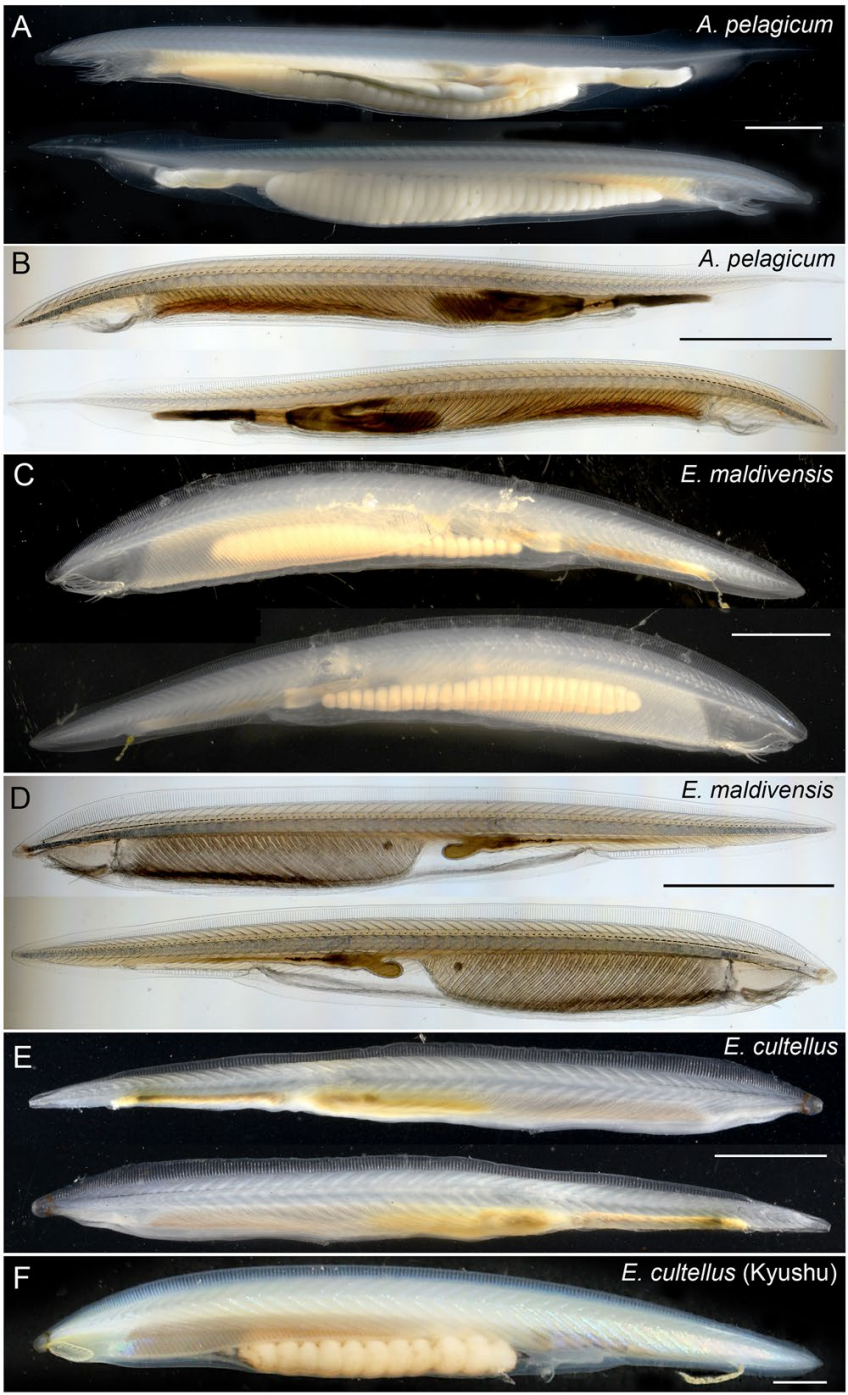
Left and right lateral views of dextral gonad amphioxus species. (**A**) Live *Asymmetron pelagicum* from Nanwan Bay under incident light. (**B**) Live *A. pelagicum* without gonads from Nanwan Bay under transmitted light. (**C**) Live *Epigonichthys maldivensis* from Nanwan Bay under incident light. (**D**) Live *E. maldivensis* without gonads from Nanwan Bay under transmitted light. (**E**) Fixed *E*. *cultellus* from the South China Sea under incident light. (**F**) Lateral view of live *E. cultellus* from northern Kyushu under incident light. Scale bars 2 mm.

For *Epigonichthys* specimens, mitogenomes obtained from four specimens from Nanwan Bay clustered as a clade with a mitogenome of *E. maldivensis* from Okinawa, Japan (Fig. 1), and the gene order was identical to that of *E. maldivensis* ^3^. However, the lengths of nucleotide sequences slightly varied from 14,967 to 14,970 bp (AP015025-015028) due to indels as seen in the *Asymmetron* mitogenomes. A previous study on the complete mitogenome of an *Epigonichthys* specimen from the South China Sea has clarified its difference at the species level from *E. maldivensis* from Okinawa, Japan^17^, and therefore we examined the numbers of myomeres, gonads, and dorsal and preanal finboxes of *Epigonichthys* specimens from Nanwan Bay and the South China Sea (Table 1). These data reconfirmed clear morphological differences between the specimens from Nanwan Bay and the South China Sea (Fig. 5). Previously, based on morphometric data, the specimens from the South China Sea were assigned to *E. cultellus* ^17^ while specimens from Nanwan Bay were assigned to *E. maldivensis* ^14, 16^. Our results showed that whole mitogenomic sequences of *Epigonichthys* from Nanwan Bay are most similar to and had gene orders identical to that of *E. maldivensis* from Okinawa and reconfirm that the *Epigonichthys* species found sympatrically with *Asymmetron* species in Nanwan Bay is *E. maldivensis*.

**Table 1:** Metrical characteristics of *Epigonichthys* species from China-Taiwan-Japan waters.

Specimens	Body length (mm)	No. of myomeres	No. of dorsal finboxes	No. of preanal finboxes	No. of gonads
*Em*@Taiwan #1	15.2	66	320	34	25
*Em*@Taiwan #2	29.0	67	294	32	23
*Em*@Taiwan #3	23.0	67	308	25	NA
*Em*@Taiwan #4	24.0	67	288	32	NA
*Em*@Taiwan #5	26.0	66	312	36	NA
*Em*@Taiwan #6	15.4	68	293	29	NA
*Em*@Taiwan #7	14.1	69	303	25?	NA
*Em*@Taiwan #8	13.3	58	292	20	NA
mean ± s.d.	20.00 ± 5.77	66.0 ± 3.2	301.3 ± 10.6	29.7 ± 5.1 (n = 7)	
*Ec*@China #1	15.6	51	222	NA	NA
*Ec*@China #2	14.1	47	228	NA	NA
*E* sp@Kyushu #1	30.0	46	202	NA	11
*E* sp@Kyushu #2	NA	50+	230	20	15

*Em*, assigned to *Epigonichthys maldivensis* ^16^; *Ec*, assigned to *E. cultellus* ^17^. Mean ± s.d. with blue highlight is for *E. maldivensis* from Nanwan Bay, Taiwan.

### Identity of *Epigonichthys*-like specimens from temperate waters in Kyushu, Japan

In Japan, a single *Epigonichthys* specimen was previously recorded from the coastal waters of Shirahama, Wakayama (33°42′N 135°18′E), representing the most northern record of the genus *Epigonichthys*, and this specimen was assigned to *E. maldivensis* based on morphometric data^31^. In this study we collected two specimens morphologically distinct from *E. maldivensis* (Fig. 5) from northern Kyushu (Fukuoka, 33°47′40.3″N 130°24′25.1″E; Ariake Sea, 32°32′09″N 130°19′10″E) during field collections between 2001 to 2014. The collection sites are located at almost the same latitude as Shirahama, and similarly influenced by the Kuroshio Current. The morphometric data of the Kyushu specimens (Table 1) suggested an affinity to *E. australis* (Raffe, 1912)^7^ or *E. cultellus*, and the complete mitogenome was 14,985 bp in length (AP015024) and almost identical to that of *E. cultellus* from the South China Sea [99.73% (40/14,985 substitution sites) similarity] with identical gene order^17^. We thus assigned these *Epigonichthys*-like specimens from Kyushu to *E. cultellus*, although their body sizes were 1.5 times larger than the mean size of specimens from the South China Sea^17^.

## Discussion

### Generic phylogeny and evolutionary history

Our analyses of mitogenomic sequence data reconfirm the previously observed divergence pattern of the cephalochordate lineage, in which *Asymmetron* diverged first, followed by *Epigonichthys* and *Branchiostoma* (Fig. 1). Species within the *Asymmetron* clade are less divergent compared with those in the *Branchiostoma* + *Epigonichthys* clade. In particular, the close relationship between the Pacific and Atlantic *Asymmetron* populations is remarkable. To explain the genetic divergence within *Asymmetron*, it has been suggested that *Asymmetron* originated in the eastern Tethys Sea during the breakup of Pangaea in the Mesozoic, then separated into Indo-West Pacific and Atlantic populations, and finally the Atlantic population expanded into the West Pacific from the Atlantic to become sympatric with the Indo-West Pacific population as found in the Kuroshima population^4^. However, this scenario is not without weak points. We found no Indian haplotype of *coxI* in Taiwanese specimens (n = 25). Furthermore, whole mitogenomic sequence comparisons separate the Indian group from the Pacific + Atlantic group but not the Atlantic group from the Indian + West Pacific group. If gene flow is directly ongoing between Indian and Japanese populations as suggested previously^4^ or via an intermediate population on the eastern coast of Australia (Indian haplotypes of *coxI* have been reported from this region^4^), we would expect to find the Indian haplotype in the Taiwanese population as currents from the northern South China Sea seasonally join the Kuroshio Current via the Luzon Strait, onto which Nanwan Bay opens^32^. Additionally, the Kuroshio Current passes through this strait seasonally^32^. However, we did not find any Indian haplotype in Nanwan Bay, and this absence is consistent with proposed Cenozoic currents that flowed from the Pacific to the Indian Ocean with no opposite direction flow except at high latitudes^33^. Additionally, in the Cenozoic there was no northward current along the eastern coast of Australia^34^. We thus speculate that the Indian *coxI* haplotype found in the Okinawan population may be due to recent human introduction, as has been seen with the introduction of other marine species via the discharge of ballast water^35^.

The mitogenomic analyses suggest that the *Asymmetron* clade after the divergence of *A. inferum* was present in the Indian Ocean and migrated westward, passing through the Neo-Tethys into the Atlantic, and then into the Pacific through a seaway between North and South America. However, we could not detect any evidence of direct migration from the Indian Ocean towards the eastern coast of Eurasia. The genetic proximity between the Pacific and Atlantic populations suggests that gene flow between these two groups was not terminated until the formation of the Isthmus of Panama. The formation of the isthmus has been suggested to have been a long and complex process, but many marine taxa living in shallow waters have divergence times between Caribbean and Pacific groups between 1.03 to 4.35 Ma^36, 37^. The formation of the isthmus and the closure of the Neo-Tethys, estimated as 14 Ma^38^, appear to have caused species diversification within *Asymmetron* as our divergence timing estimates corresponded well to these geological events.

The absence of *Epigonichthys* from the Atlantic is interesting. When we take into account the fact that *E. maldivensis* is sympatric with the Pacific population of *Asymmetron* and that some *Epigonichthys* species are distributed in temperate regions such as the Bass Strait in southern Australia (annual water temperatures 12–22 °C^39^) and northern Kyushu, Japan (14–28 °C), it seems unlikely that *Epigonichthys* was once distributed in the Atlantic and has since gone extinct. The diversity of extant *Epigonichthys* species in Oceania^7^ suggests that the origin of the *Epigonichthys* lineage may be in this region. On the other hand, the *Branchiostoma* lineage shares a common ancestor with the *Epigonichthys* lineage but is distributed worldwide. Differences in present distributions between the *Epigonichthys* and *Branchiostoma* clades may be attributable to large egg numbers in *Branchiostoma*. Larger numbers of larvae with long pelagic life like amphioxus^40^ could increase chances for wider distributions via ocean currents^41^.

### Character polarity in amphioxus lineage

**Figure 6:**
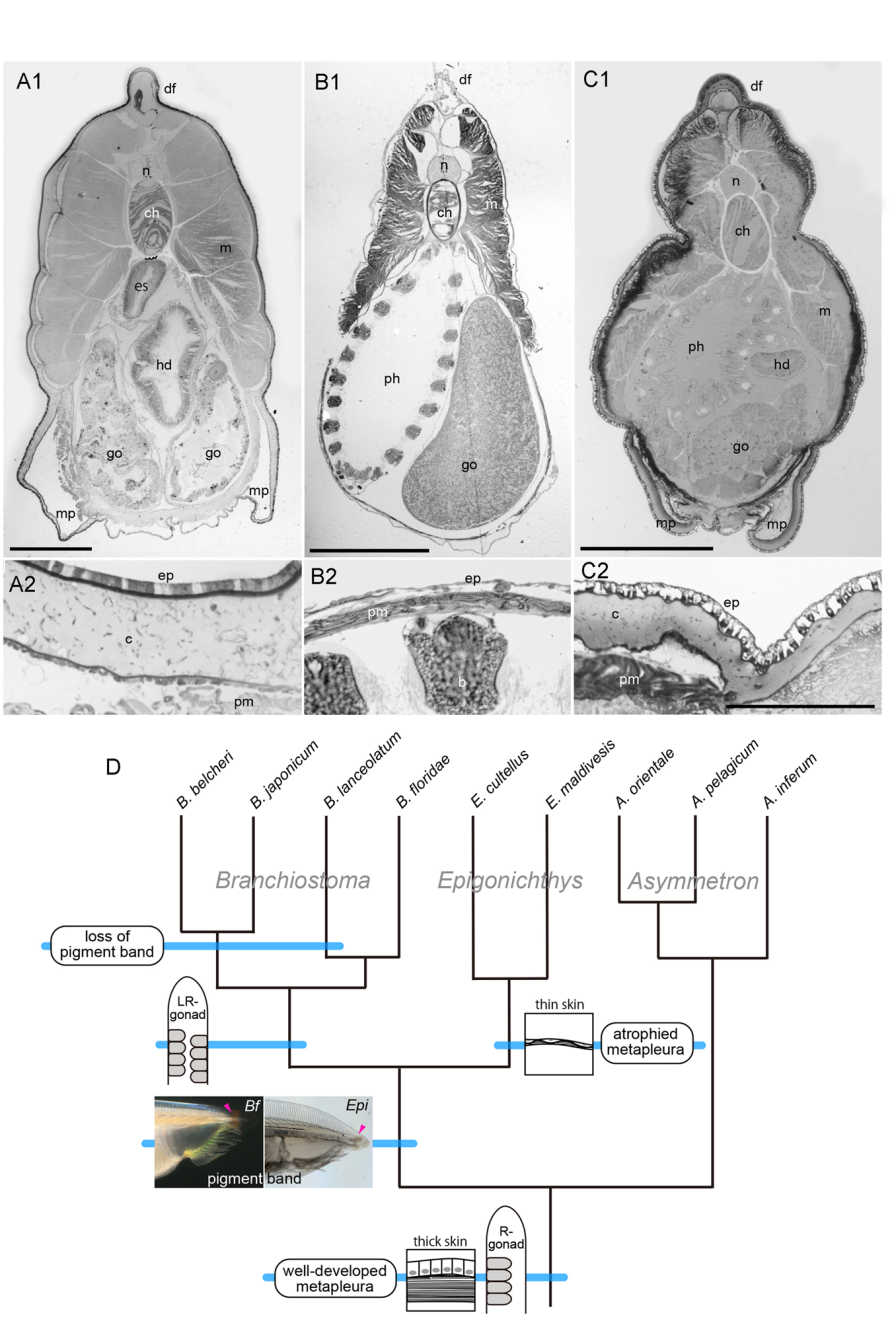
Transverse sections of three amphioxus genera and parsimoniously expected character polarity. (**A1**) Transverse section at posterior pharyngeal region of *Branchiostoma japonicum*. (**A2**) Thick epidermal epithelium and collagen layer in *B. japonicum*. (**B1**) Transverse section at pharyngeal region of *Epigonichthys maldivensis*. (**B2**) Squamosal epidermal epithelium and very thin collagen layer in *E. maldivensis*. (**C1**) Transverse section at posterior pharyngeal region of *Asymmetron pelagicum*. (**C2**) Cuboidal epidermal epithelium and well-developed collagen layer. (**D**) Metapleura, thick skin, and dextral gonads are ancestral characters in amphioxus lineage. Genus *Epigonichthys* displays most derived features. Branching pattern in *Branchiostoma* clade follows nucleotide-based trees. b, branchial bar; *Bf*, *Branchiostoma floridae*; c, collagen layer; ch, notochord; df, dorsal fin; ep, epidermis; *Epi*, *Epigonichthys*; es, esophagus; go, gonad; hd, hepatic diverticulum; m, myomeric muscle; mp, metapleuron; ph, pharynx; pm, pterygial muscle. Scale bars 0.5 mm for A1, B1, C1, and 0.2 mm for C2 applicable to A2, B2.

There is a large time gap between the origin of the cephalochordate lineage, which likely occurred in the Precambrian, and the diversification into extant taxa in the Cenozoic, and therefore it is difficult to ascertain the form of the LCA of the amphioxus crown lineage. However, the character polarity of major traits deduced from the phylogenetic tree may provide insights into the LCA of extant amphioxus taxa. The most conspicuous feature of the amphioxus lineage is left-right asymmetrical development. Gonad development is also asymmetrical, with *Asymmetron* and *Epigonichthys* sharing dextral gonads while *Branchiostoma* spp. develop gonads on both sides (Fig. 6). Dextral gonads are thus shared by the two major clades, which supports the evolution from ancestral unilateral to derived bilateral gonads^3^. This polarity suggests that the asymmetrical development of amphioxus is tightly related to the origin of this lineage as there are no comparable outgroups, and this idea has also been supported by a comparative developmental study^42^. In amphioxus, the most pronounced left-right asymmetry occurs in the larval body^1^ with some variations between genera; *Asymmetron* larvae display less-pronounced left-right asymmetry in the location of the primary gill openings (future left gill openings) and anus compared to *Branchiostoma* larvae^43^.

Molecular studies have demonstrated that amphioxus larval asymmetry is controlled by the Nodal-Pitx signaling unit in early development^44^, especially in oropharyngeal development, in which the asymmetry is most pronounced^45^. Nodal signaling also controls apoptosis of germ cells in sea urchins^46^. These studies suggest that Nodal signaling and its downstream gene regulatory networks may have been key players in the evolution of the amphioxus lineage, and subtle differences in these molecular functions may explain the variations in the developmental left-right asymmetry including gonad development in the amphioxus lineage.

*Branchiostoma* and *Epigonichthys* are larger than *Asymmetron* species in body size, and have similar appearances, sharing preanal finboxes, caudal myomeres, and head patterns with similar buccal cirri and six myomeres anterior to the velum. Further, *B. floridae* shares a pigment band at the base of the rostrum with *Epigonichthys* species (Fig. 6). These features support the monophyly of these two genera as recovered in the phylogenetic analyses. In contrast, the skin of *Epigonichthys* is specialised thin squamous epithelium with a thin subepidermal collagenous layer, contrary to the cuboidal epithelium with a thick collagenous layer in *Asymmetron* and *Branchiostoma* (Fig. 6). Other unique conspicuous features of *Epigonichthys* are a dorso-ventrally expanded flat body with tall dorsal finboxes (Fig. 5), and atrophied metapleura (longitudinal folds that develop on both sides of the belly in the other two genera), that are particularly noticeable when gonads are developing (Fig. 6), as well as a slight leftward shift of the buccal opening (the amphioxus mouth is located at the end of buccal cavity separating it from the pharynx and is called the ‘velum’) (Fig. 5). These features suggest that *Epigonichthys* is the most derived group among extant amphioxus lineages.

All extant amphioxus clades do not develop a mucus or cuticle layer on the epidermis, and have a naked simple epithelium despite inhabiting sandy substrata (Fig. 6). These unique features are thus likely an ancestral character in the lineage. *Asymmetron* and *Branchiostoma* embryos and larvae move with monocilia that develop throughout the epidermis similar to cnidarian planula larvae even after they develop locomotive myomeres^43, 47^, and the cilia disappear during metamorphosis when larval length reaches approximately five mm in *B. japonicum* ^40^. Although *Branchiostoma* larvae are regarded as pelagic, they frequently slide by ciliary movement on the bottom when maintained in culture tanks^48^, suggesting that this small ciliated stage is not necessarily pelagic. The naked epidermal surface of extant amphioxus taxa may be a retained feature of their ciliated ancestral form as deduced from the fact that most ciliate larvae found across animal phyla do not develop a thick mucus layer on the epidermis as the mucus negatively influences locomotion and feeding. It should be noted that some animal larvae develop epidermal mucus cells to secret mucus strands for collecting food particles or locomotion control^49, 50^.

We estimated the diversification into the three extant clades occurred in the Eocene (38.8–46.0 Ma with 33.9–56.6 Ma 95% confidence interval). These estimates are more recent than compared to previous estimates^3–5, 10^. Additionally, they are consistent with the genetic proximity between species observed within the *Asymmetron* clade, and also with the recent finding that amphioxus displays a rapid evolutionary rate comparable to that of vertebrates^13^. The estimates for species splits within the *Asymmetron* clade likely correspond with geological events that occurred onward from the Miocene, such as the closure of the Neo-Tethys^38^ and the formation of the Isthmus of Panama^36, 37^. Given the Precambrian origin of the amphioxus lineage, the amphioxus lineage survived many mass extinction events before its most recent diversification. Taking into account this and the character polarities mentioned above, one possible scenario for the long evolutionary history until diversification is that soon after separation from the main chordate lineage, the amphioxus ancestor acquired its modern developmental pattern with metamorphosis, and diversified as sandy bottom dwellers as seen in extant amphioxus clades. All but one ancestral group went extinct, and the surviving species then diversified into the extant clades. In this case, the cephalochordate LCA might have grown to the size of modern amphioxus. For example, the Cambrian fossils *Haikouella* ^27, 51^ and *Pikaia* ^28^, which may have had affinity to the amphioxus lineage, had body sizes comparable to or larger than those of modern amphioxus. However, the fact that many animal groups rapidly increased their body sizes by the Middle Cambrian^52^ suggests another possibility; that the LCA of chordates was comparatively tiny and that the common ancestor of the extant amphioxus lineage retained this tiny size until the most recent diversification. This idea seems more likely when considering the character polarity found in extant species, especially the naked simple epithelial epidermis without mucus coat that is found in all amphioxus clades. This idea is also consistent with the small-sized ancestral state expected from the polarity of the body size, in which the *Asymmetron* clade has smaller sizes than the *Branchiostoma* clade, and the *Epigonichthys* clade displays a variety of sizes.

We hypothesise, therefore, that the LCA of extant amphioxus had a few-mm long ciliated body with left-right asymmetry, and that diversification into extant clades may have been triggered by increasing body size and the acquisition of metamorphosis. The LCA thus may have retained the larva-like anatomy of extant amphioxus with unilateral gonads developing near the mid-ventral region (anatomically derived from the right coelom). Given the small body size of the proposed LCA and a likely limited number of eggs [egg sizes of 120–140 μm do not vary among extant taxa, suggesting an ancestral character (for *A. pelagicum*, ref. 43)], the proposed tiny LCA might have inhabited shoals on the surface of fine sandy seafloors.

### Mitogenomic systematics and population identities

The genus *Asymmetron* currently comprises two species, *A. lucayanum* and *A. inferum*. The former species is distributed circumtropically and proposed to contain at least three cryptic species^4^. Our phylogenetic analyses reconfirm the existence of these three clades; Indian, Pacific and Atlantic Ocean groups. Although these clades have been proposed to be three species^4^, it is reasonable to group the Pacific and Atlantic clades into a single species based on a lack of sequence divergence (~7%; interspecific differences in *Epigonichthys* and *Branchiostoma* are ~25% in nt comparison), and consider the Indian Ocean clade as a different species (~23% compared with the Pacific or Atlantic clade in nt). As the Pacific + Atlantic *Asymmetron* clade included haplotypes from Hawai’i (Fig. 4 and ref. 4), we propose that *Asymmetron pelagicum* Günther, 1889 described from Hawai’i should be used as the correct binomial name for the Pacific + Atlantic clade, replacing the junior synonym *Asymmetron lucayanum* Andrew, 1893, described from the Bahamas. If, in the future, researchers decide the Atlantic clade should be split from *A. pelagicum*, then the name *A. lucayanum* should be utilised for this species-group. Similarly, specimens from the Maldives in the Indian Ocean were described as *Asymmetron orientale* Parker, 1904, and we propose this binomial should be used for the Indian clade of the *A. lucayanum* complex. The number of *Asymmetron* species in the world is, therefore, at least three, including the anaerobic sulfidophilic *A. inferum* ^2^. The *Asymmetron* specimens from Nanwan Bay at the southern tip of Taiwan are assigned to *A. pelagicum* (=former *A. lucayanum*) based on the present mitogenomic analyses and morphometric data.

The genus *Epigonichthys* has been reported from the eastern coast of African continent to Hawai’i, and is not present in the Atlantic^7^. This genus currently comprises six species, most of which are distributed in Oceania^7^, but taxonomically many questions remain. Previous studies on *Epigonichthys* from Taiwan have assigned specimens to *E. maldivensis* based on morphometric data^14, 16^. Our mitogenomic sequence analyses showed a close similarity of Taiwanese *Epigonichthys* to *E. maldivensis* from Okinawa, supporting the assignment of Taiwanese *Epigonichthys* specimens to *E. maldivensis* for now, although molecular examination of specimens from the Maldives are needed to confirm this.

On the other hand, the *Epigonichthys* specimens from northern Kyushu, Japan, are morphologically distinct from *E. maldivensis* specimens from Taiwan. The mitogenomic sequence of this rare amphioxus was almost identical to that of *E. cultellus* from the South China Sea^17^, and we have identified these specimens to this species. Although the very low numbers of specimens of this amphioxus in Japan raises the question of whether the species breeds at the collecting sites or not, this study confirms that two species of *Epigonichthys*, *E. maldivensis* and *E. cultellus*, are present in Japanese coastal waters. To further understand the *Epigonichthys* clade, studies on Oceanian populations that lack modern analytical data are needed.

## Methods

### Animal sampling and animal care

Amphioxus (n = 83) with dextral gonads were collected from three sites (21°57′12.8″N 120°46′06.2″E, 21°57′11.1″N 120°46′08.5″E, 21°57′11.5″N 120°46′06.7″E) in Nanwan Bay, Taiwan, by SCUBA diving with collecting bags in 2014 and 2015 (permission No. 1032900823 issued by Kenting National Park Headquarters, Taiwan) (Supplementary Fig. S1). Two specimens were collected from *Branchiostoma* habitats in northern Kyushu, Japan, one in Hakata Bay (33°47′40.3″N 130°24′25.1″E), Fukuoka, in 2012, and the other in the Ariake Sea (32°32′09″N 130°19′10″E), Kumamoto, in 2008 (no permission required for amphioxus collection). DNA from a specimen collected in Bimini Lagoon, the Bahamas (25°43′22.7″N 79°17′38.0″W) (courtesy of Dr. Jr-Kai Yu) was also utilised (Supplementary Fig. S1). Specimens from Nanwan Bay were maintained in the laboratory and individuals damaged during culture were fixed with 4% paraformaldehyde in 0.1 M 3-(N-morpholino) propanesulfonic acid (MOPS) buffer (pH 7.5) and then stored in 75% ethanol or directly fixed with 75% ethanol. The specimens from Fukuoka were fixed with the same fixatives in the field. All fixed specimens were stored at −20 °C until use. *Branchiostoma japonicum* specimens for histological observations were collected from a laboratory colony^53^, and *Epigonichthys cultellus* specimens used for morphometrical data were collected from off Guandong, China^17^. All animals in the present study were maintained in accordance with guidelines established by Hiroshima University for the care and use of experimental animals. Our experimental protocols were approved by the Hiroshima University Animal Research Committee (Approval number: G14-2.). Animals were fixed at the laboratory under anaesthesia with 0.02% amino benzoic acid ethyl ester in millipore-filtered seawater.

### DNA extraction, amplification, and sequencing of short DNA fragments

DNA was extracted individually from 29 *Asymmetron* specimens from Nanwan Bay and 5 *Epigonichthys* specimens from Nanwan Bay and Hakata Bay by using NucleoSpin Tissue (MACHEREY-NAGEL, Germany). For the Nanwan Bay *Asymmetron* specimens, PCR was performed initially to amplify a fragment of the *cytochrome c oxidase subunit I* (*coxI*) gene with the primer set AmphL109/AmphH1325^4^. The PCR was carried out with genomic DNA as template (95 °C for 3 min, 95 °C for 30 sec x35, 55 °C for 30 sec, 72 °C for 30 sec). The amplicons were directly sequenced as described previously^54^. Amplification of two DNA fragments that cover the full length of the mitogenomic DNA excluding the *cox1* region was performed by PCR (95 °C for 3 min and 95 °C for 30 sec x35, 68 °C for 15 min) with primer sets shown in Supplementary Table S1. The sizes of amplicons were confirmed by gel electrophoresis, and the DNA fragments in the gel blocks were purified using FastGene Gel/PCR Extraction Kit (Nippon Genetics, Japan).

### Whole mitogenomic DNA sequencing

Whole mitogenomic DNA was sequenced using two long DNA fragments with Ion Torrent PGM™ (Thermo Fisher Scientific, MA). The two fragments had adapters with different barcode sequences attached (Ion Xpress Barcode Adapters) that were mixed individually and used as templates for constructing an amplicon library with NEBNext Fast DNA Fragmentation & Library Prep Set (New England Biolabs, MA) for Ion Torrent. The libraries constructed were quantified by using KAPA Library Quantification Kits (KAPA Biosystems, MA) for Ion Torrent and pooled into a single tube. The pooled library sample was further amplified by an emulsion PCR with Ion PGM Template OT2 200 Kit (Thermo Fisher Scientific, MA). The product was finally sequenced on an Ion PGM sequencer (Thermo Fisher Scientific, MA) with Ion 318 Chip Kit v. 2 and Ion PGM Sequencing 200 Kit v. 2.

### Phylogenetic analyses

Haplotypes of partial segments of *cytochrome c oxidase I* (*coxI*) from 26 specimens of *Asymmetron* species sequenced in this study, and 51 unique haplotypes of the same *coxI* segments from 80 specimens were identified^4^. A neighbour-joining (NJ) tree was constructed under the p-distance^55^ within MEGA 5.2 based on the sequences of *coxI*. Phylogenetic trees based on maximum likelihood (ML) with T92 + G models^56^ (ML model selected after model selection analysis) and Bayesian inference (BI) were also constructed by the method mentioned below. Bootstrap values were calculated with 1,000 pseudoreplicates. A minimum spanning network was also constructed using TCS 1.21^57^. The maximum number of steps to connect haplotypes parsimoniously was calculated with a 99% limit.

From 12 full-length mitogenomic sequences obtained in this study (7 *Asymmetron*, 1 *E. cultellus*, and 4 *E. maldivensis* individuals) and 10 mitogenomes from GenBank (Supplementary Table S2), we prepared two subsets of data: amino acid (aa) sequences of 13 protein genes, and all nucleotide (nt) sequences of the mitogenome excluding the control region. Both datasets were aligned separately for each gene by using clustalW^58^ within MEGA 5.2^59^ with default settings. For protein coding genes, we first aligned aa and then aligned the nt correspondingly to their codons. Poorly aligned regions and gap sites were deleted by using gBlocks v. 0.91b^60^ while specifying the type of sequence.

We also constructed an aa sequence dataset from 952 orthologous protein coding nuclear genes of *Branchiostoma belcheri*, *B. lanceolatum*, *B. floridae*, *Asymmetron lucayanum*, and four vertebrate species (*Danio rerio* ^61^, *Xenopus tropicalis* ^62^, *Mus musculus* ^63^, and *Homo sapiens* ^64^). The aa sequences of four vertebrates (GRCz10, JGI 4.2, GRCm38.p4, and GRCh38.p7) were retrieved from Ensembl Release 85 (July 2016) and those of *B. belcheri* (v.18h27.r3) and *B. floridae* (v.1.0) were retrieved from each portal site of genome sequencing project (http://genome.bucm.edu.cn/lancelet/ and http://genome.jgi.doe.gov/Brafl1/Brafl1.home.html). The aa sequences of *B. lanceolatum* were deduced from a published transcriptome^65^ by using TransDecoder 3.0.0 (https://transdecoder.github.io/). For *A. lucayanum*, raw RNA-seq reads^10^ were retrieved from the DDBJ Sequence Read Archive (DRA) and transcriptome data was assembled by using Trinity v. 2.1.1 after quality control of the reads^10^. The transcriptome data of *A. lucayanum* and *B. lanceolatum* were then translated into aa sequences. Orthologous 952 gene sets were also constructed by using Proteinortho (v. 5.12) and the PhyloTreePruner pipeline^10^.

Phylogenetic trees were constructed by ML and Bayesian (BI) methods based on the nuclear transcriptome and whole mitogeninic sequences. To select the optimum substitution models for each gene, Aminosan and Kakusan4^66^ were used based on the Akaike information criterion (AIC). ML analyses were conducted using RAxML v. 8.1.24^67^, evaluated by bootstrap values of 1,000 pseudoreplicates. BI analyses were conducted using MrBayes 5D^68^ with two independent runs of four Markov chain Monte Carlo (MCMC) chains. Analyses were run for ten million generations, and trees were sampled every 1,000 generations. Convergence among runs was verified by examining the likelihood plots using Tracer 1.6^69^. The first 50% of trees were discarded as burn-in and the remaining trees were summarized with posterior probabilities at the nodes.

### Inference of divergence time

We estimated divergence times based either on nuclear transcriptomes with outgroup reference points or mitogenomes with ingroup reference points. First we used aa sequences derived from nuclear transcriptomes with calibration points at the cephalochordate-vertebrate (550.0 ± 16.0 Ma)^25^, osteichthyan-tetrapode (419.0 ± 1.4 Ma)^25^, anamniote-amniote (340.0 ± 5.0 Ma)^24^ splits, and rodent-primate split (81.0 ± 10.0 Ma)^24^. For the mitogenomic sequences, we excluded 22 tRNAs and applied ingroup calibration points obtained from the first estimate based on the nuclear transcriptomes; *Branchiostoma-Asymmetron* (46.0 ± 5.5 Ma), *B. belcheri*-(*B. lanceolatum* + *B. floridae*) (28.2 ± 5.5 Ma), and *B. lanceolatum*-*B. floridae* (22.6 ± 2.3 Ma). Divergence times were estimated by using BEAST 1.8.4 with the random local clock model that assumes rate changes across branches^70^. The substitution model used in the analysis was selected in Kakusan4 under AIC according to each codon position. For the mitogenome-based estimation, we also applied geological calibration points to the split between the Pacific and Atlantic populations of *A. pelagicum* (=former *A. lucayanum*) (2.06 Ma: 1.03–4.35 Ma = 95% credible interval (CI))^36, 37^ and to the split between the Indian and Atlantic populations (14 Ma: 6.16–21.84 Ma = 95% CI)^38^. Tree topology was fixed to the ML tree and prior distributions of the time of the most recent common ancestors of these species were constrained by normal distributions to cover the 95% CIs arbitrarily. All other model parameters were set to default priors. For MCMC analysis, we performed a run of 10 million generations, sampling every 1,000th generation and removing the initial 10% of samples as burn-in. Convergence of the chains was confirmed using Tracer v. 1.6^69^.

### Morphometric analyses

Live or fixed specimens were photographed under microscope or digital camera (D800 Nikon, Japan) with a close-up lens. Digital photographs were visually optimized by using Photoshop CS6 (Adobe, CA), and body length, as well as the numbers of dorsal finboxes, preanal finboxes, gonads, and myomeres were measured or counted from images.

### Histological sectioning

Small specimens with gonads of *Asymmetron pelagicum* (=former *A. lucayanum*), *Branchiostoma japonicum*, and *Epigonichthys maldivensis* were cut into anterior and posterior halves and fixed with 75% ethanol or 4% paraformaldehyde in 0.1 M MOPS buffer (pH 7.5) with 0.5 M NaCl at 4 °C overnight. After washing with Millipore filtered seawater (MFSW), they were stained with 1% tannic acid in MFSW for 2 hours, washed again with MFSW, and then fixed with 1% osmium tetroxide in MFSW at 4 °C for 2 hours. The postfixed specimens were dehydrated through a graded ethanol series and embedded in hydrophilic Epon. The pharyngeal region of embedded specimens was sectioned with glass knives at 1 μm and stained with 0.1% toluidine blue containing 1% sodium borate at 60 °C.

## Electronic supplementary material

supplementary file of Igawa et al.
