# Supplementary material for: Evolutionary history of the extant amphioxus lineage with shallow-branching diversification
Source: Sci Rep. 2017 Apr 25;7:1157. doi: 10.1038/s41598-017-00786-5 (PMC5430900; doi:10.1038/s41598-017-00786-5)
Supplement: Supplementary file 1 — supplementary file of Igawa et al. [file 41598_2017_786_MOESM1_ESM.pdf]

Supplementary information

"Evolutionary history of the extant amphioxus lineage with shallow-branching diversification" by Takeshi Igawa, Masafumi Nozawa, Daichi G. Suzuki, James D. Reimer, Arseniy R. Morov, Yiquan Wang, Yasuhisa Henmi, and Kinya Yasui

Table S1. Primers and their combinations for amplification of mitochondrial genomes.
Table S2. Samples and their mitochondrial genomes used in this study.
Figure S1. Map of collecting localities for newly collected *Asymmetron* and *Epigonichthys* specimens.
Figure S2. Divergence time estimates based on mitogenomic nucleotide sequences in amphioxus lineage with geological calibration points.

Table S1. Primer and their combinations for amplification of mitochondrial genomes

Primer	Sequence (5' to 3')	Reference
AmphL109	ATTCGNGCNGAAYTNTCNCAGCC	ref. 1
AmphH1325	TCNGAATAYCGNCGWGGTATNCC	ref. 1
AmphLAL1076-cox1	CTGTAGGTGGGTTAACAGGAATTGTATTAGC	ref. 2
EmLAL1076-cox1	CCGTGGGTGGTTTAACGGGAATTGTATTAGC	this study
EcLAL1076-cox1	CTGTGGGGGGTTTAACTGGAATCGTGCTAGC	this study
AmphLAH12317-rrnL	CTAGGAATCTTGATCCAACATCGAGGTCGC	ref. 2
AmphLAL11918-rrnL	GACTGTGCAAAGGTAGCATAATCACTTGCCC	ref. 2
AmphLAH916-cox1	CCTGTTGGTACAGCAATTACTATAGTAGCAGC	ref. 2
AlLAH916-cox1	CCTGTGGGGACGGCAATAACTATAGTTGCAGC	this study
EcLAH916-cox1	CCGGTAGGTACAGCAATTACCATCGTAGCCGC	this study
**Species**	**Fragment 1 (*coxI - 12S rRNA*)**	**Fragment 2 *(12S rRNA -coxI*)**
Asymmetron lucayanum	AmphLAL1076-cox1 / AmphLAH12317-rrnL	AmphLAL11918-rrnL / AlLAH916-cox1
Epigonichthys maldivensis	EmLAL1076-cox1 / AmphLAH12317-rrnL	AmphLAL11918-rrnL / AmphLAH916-cox1
Epigonichthys cultellus	EcLAL1076-cox1 / AmphLAH12317-rrnL	AmphLAL11918-rrnL / EcLAH916-cox1

Table S2. Samples and accession numbers of mitochondrial genomes used in this study

Species	Locality	Accession number	Reference
Asymmetron inferum	off Cape Nomamisaki, Kagoshima, Japan	AP009352	ref. 2
formerly *Asymmetron lucayanum*	off Kuroshima Island, Okinawa, Japan	AB110092	ref. 3
	Castle Harbour, Bamuda	AP009354	ref. 2
	Nanwan Bay, Taiwan	AP015017	this study
	Nanwan Bay, Taiwan	AP015018	this study
	Nanwan Bay, Taiwan	AP015019	this study
	Nanwan Bay, Taiwan	AP015020	this study
	Nanwan Bay, Taiwan	AP015021	this study
	Nanwan Bay, Taiwan	AP015022	this study
	Bimini Lagoon, the Bahamas	AP015023	this study
Epigonichthys cultellus	off eastern Guandong, China	KC896827	ref. 4
	Hakata Bay, Fukuoka, Japan	AP015024	this study
Epigonichthys maldivensis	off Kuroshima Island, Okinawa, Japan	AB110093	ref. 3
	Nanwan Bay, Taiwan	AP015025	this study
	Nanwan Bay, Taiwan	AP015026	this study
	Nanwan Bay, Taiwan	AP015027	this study
	Nanwan Bay, Taiwan	AP015028	this study
Branchiostoma belcheri	Xiamen waters, China	AY932825	ref. 5
Branchiostoma japonicum	Hakata Bay, Fukuoka, Japan	AB083383	ref. 6
Branchiostoma lanceolatum	Helgoland, Germany	AB194383	ref. 3
Branchiostoma floridae	Tampa Bay, USA	AB478574	ref. 6

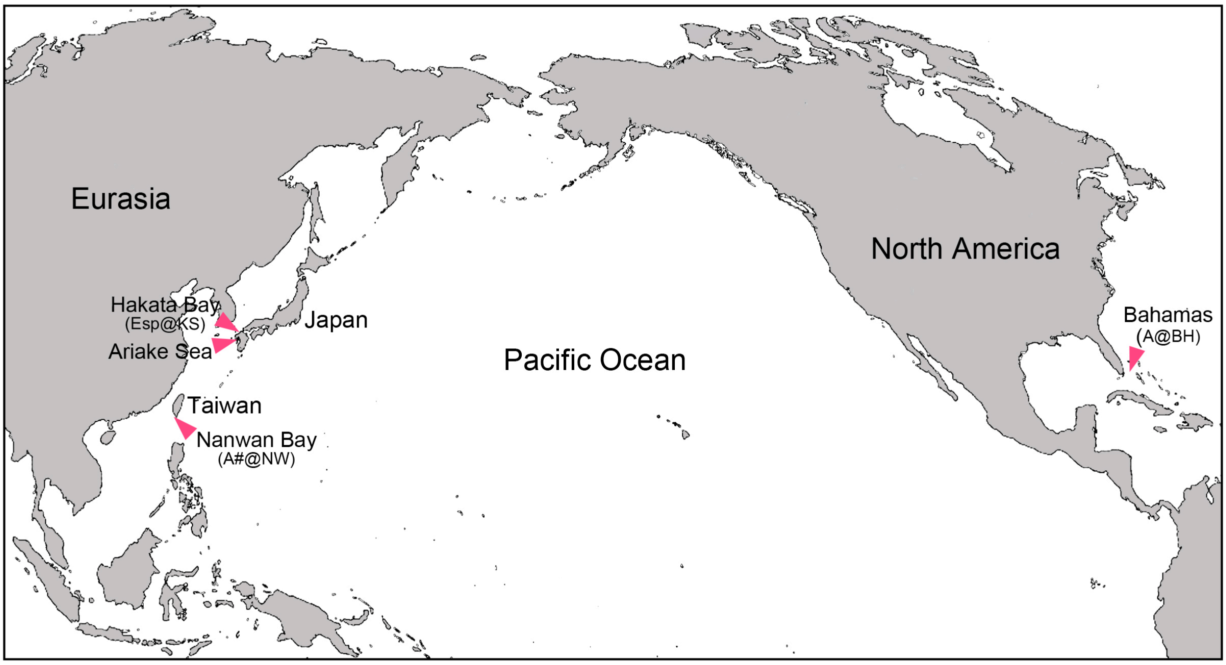

Figure S1. Map of collecting localities for newly collected *Asymmetron* and *Epigonichthys* specimens. The map was drawn based on free online map (http://gpscycling.net/fland/map/pj/07_ecker.gif) with Adobe Photoshop ver. 13.0.6 x64 and Illustrator ver. 11.0.4.

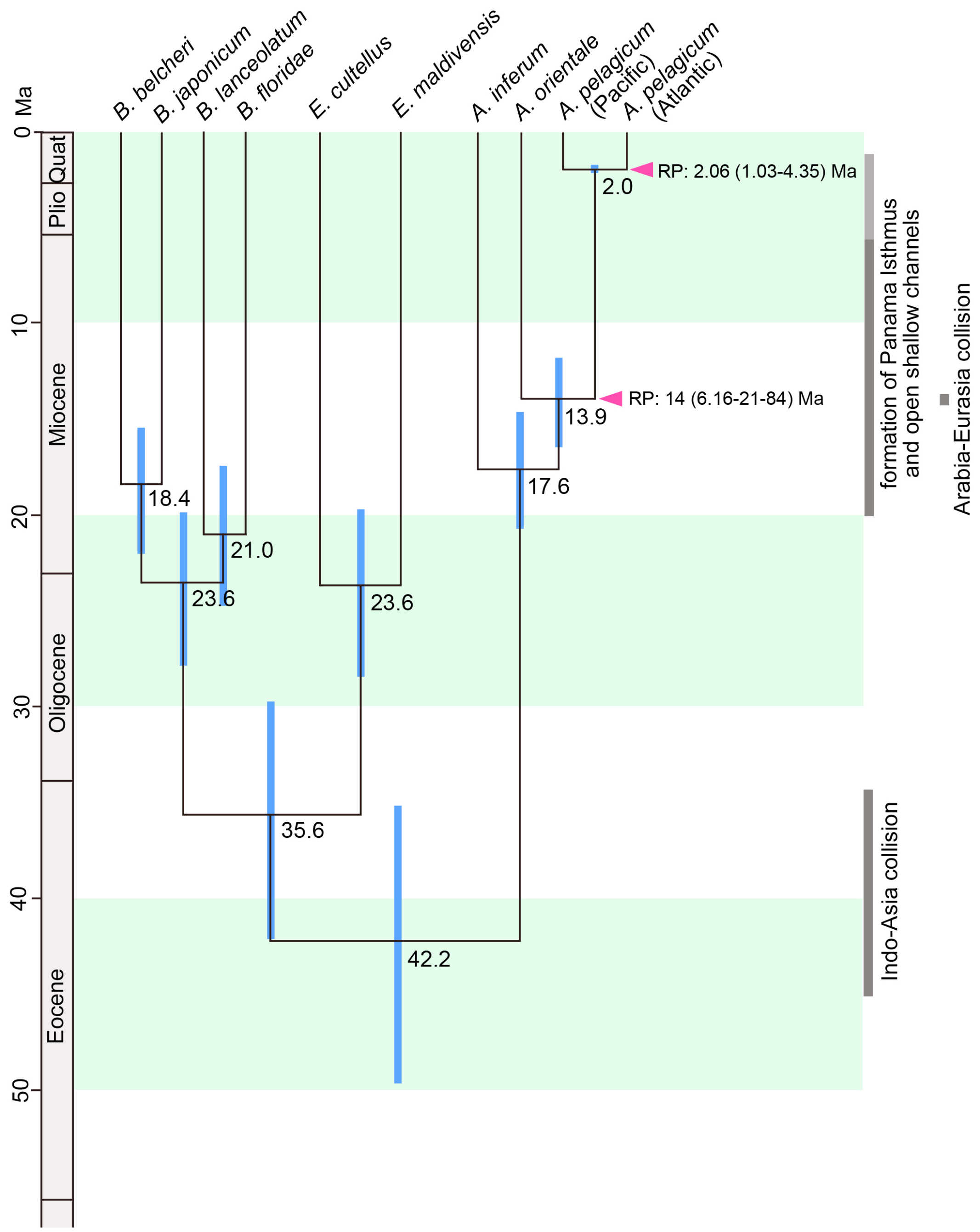

Figure S2. Divergence time estimates based on mitogenomic nucleotide sequences in amphioxus lineage with geological calibration points. BEAST 1.8.2 with the random local clock model was used with two calibration points (completion of Panama Isthmus at 2.06±0.1 Ma [7] and Arabia-Eurasia collision at 14±4.0 Ma [8].) Numerals at nodes denote estimated split time in Ma. Blue bars denote 95% confidence interval. The age of the Indo-Asia collision is from refs. [9,10].

References

1. Kon, T., Nohara, M., Nishida, M., Sterrer, W. & Nishikawa, T. Hidden ancient diversification in the circumtropical lancelet *Asymmetron lucayanum* complex. *Mar Biol* **149,** 875–883 (2006).
2. Kon, T. *et al.* Phylogenetic position of a whale-fall lancelet (Cephalochordata) inferred from whole mitochondrial genome sequences. *BMC Evol Biol* **7,** 127 (2007).
3. Nohara, M., Nishida, M., Miya, M. & Nishikawa, T. Evolution of the mitochondrial genome in cephalochordata as inferred from complete nucleotide sequences from two *Epigonichthys* species. *J Mol Evol* **60,** 526–37 (2005).
4. Li, W.-Y., Fang, S.-H. & Wang, Y.-Q. Complete Mitochondrial Genome of *Epigonichthys cultellus* (Cephalochordata: Branchiostomatidae). *Zool Sci* **31,** 766–72 (2014).
5. Zhong, J. *et al.* Complete mitochondrial genomes defining two distinct lancelet species in the West Pacific Ocean. *Mar Biol Res* **5,** 278–285 (2009).
6. Takada, Y. & Imai, T. Comparative genomics of mitochondrial DNA in *Branchiostoma* species. *J Anal Bio-Science* **32,** 167–180 (2009).
7. Bacon, C. D. *et al.* Biological evidence supports an early and complex emergence of the Isthmus of Panama. *Proc Natl Acad Sci* **112,** 6110–6115 (2015).
8. Hamon, N., Sepulchre, P., Lefebvre, V. & Ramstein, G. The role of eastern tethys seaway closure in the middle miocene climatic transition (ca. 14 Ma). *Clim Past* **9,** 2687–2702 (2013).
9. Aitchison, J. C., Ali, J. R. & Davis, A. M. When and where did India and Asia collide? *J Geophys Res* **112,** B05423 (2007).
10. Ali, J. R. & Aitchison, J. C. Gondwana to Asia: Plate tectonics, paleogeography and the biological connectivity of the Indian sub-continent from the Middle Jurassic through latest Eocene (166-35 Ma). *Earth-Science Rev* **88,** 145–166 (2008).
